# The Role of Smartwatch Technology in the Provision of Care for Type 1 or 2 Diabetes Mellitus or Gestational Diabetes: Systematic Review

**DOI:** 10.2196/54826

**Published:** 2024-12-03

**Authors:** Sergio Diez Alvarez, Antoni Fellas, Katie Wynne, Derek Santos, Dean Sculley, Shamasunder Acharya, Pooshan Navathe, Xavier Gironès, Andrea Coda

**Affiliations:** 1 School of Medicine and Public Health, College of Health Medicine and Wellbeing, University of Newcastle, University Drive, Callaghan, Newcastle, 2308, Australia, 61409916949; 2 School of Health Sciences, College of Health, Medicine and Wellbeing, University of Newcastle, Newcastle, Australia; 3 Equity in Health and Wellbeing Research Program, Hunter Medical Research Institute, Newcastle, Australia; 4 Endocrinology, John Hunter Hospital, Newcastle, Australia; 5 Queen Margaret University, School of Health Sciences, Edinburgh, United Kingdom; 6 University of Gibraltar, Gibraltar, Gibraltar; 7 School of Biomedical Sciences and Pharmacy, College of Health Medicine and Wellbeing, University of Newcastle, Newcastle, Australia; 8 Central Queensland Health, Rockhampton, Australia; 9 Department of Research and Universities, Government of Catalonia–Generalitat de Catalunya, Barcelona, Spain

**Keywords:** diabetes mellitus, flash glucose monitoring, digital health, smartwatch, smartphones, mHealth, mobile health, glucose monitoring, diabetes, gestational diabetes, systematic review, smartwatch technology, blood glucose, medication adherence, self-monitoring, usability, feasibility, mobile phone

## Abstract

**Background:**

The use of smart technology in the management of all forms of diabetes mellitus has grown significantly in the past 10 years. Technologies such as the smartwatch have been proposed as a method of assisting in the monitoring of blood glucose levels as well as other alert prompts such as medication adherence and daily physical activity targets. These important outcomes reach across all forms of diabetes and have the potential to increase compliance of self-monitoring with the aim of improving long-term outcomes such as hemoglobin A_1c_ (HbA_1c_).

**Objective:**

This systematic review aims to explore the literature for evidence of smartwatch technology in type 1, 2, and gestational diabetes.

**Methods:**

A systematic review was undertaken by searching Ovid MEDLINE and CINAHL databases. A second search using all identified keywords and index terms was performed on Ovid MEDLINE (January 1966 to August 2023), Embase (January 1980 to August 2023), Cochrane Central Register of Controlled Trials (CENTRAL, the Cochrane Library, latest issue), CINAHL (from 1982), IEEE Xplore, ACM Digital Libraries, and Web of Science databases. Type 1, type 2, and gestational diabetes were eligible for inclusion. Quantitative studies such as prospective cohort or randomized clinical trials that explored the feasibility, usability, or effect of smartwatch technology in people with diabetes were eligible. Outcomes of interest were changes in blood glucose or HbA_1c_, physical activity levels, medication adherence, and feasibility or usability scores.

**Results:**

Of the 8558 titles and abstracts screened, 5 studies were included for qualitative synthesis in this review. A total of 322 participants with either type 1 or type 2 diabetes mellitus were included in the review. A total of 4 studies focused on the feasibility and usability of smartwatch technology in diabetes management. One study conducted a proof-of-concept randomized clinical trial including smartwatch technology for exercise time prescriptions for participants with type 2 diabetes mellitus. Adherence of participants to smartwatch technology varied between included studies, with one reporting input submissions of 58% and another reporting that participants logged 50% more entries than they were required to. One study reported significantly improved glycemic control with integrated smartwatch technology, with increased exercise prescriptions; however, this study was not powered and required a longer observational period.

**Conclusions:**

This systematic review has highlighted the lack of robust randomized clinical trials that explore the efficacy of smartwatch technology in the management of patients with type 1, type 2, and gestational diabetes. Further research is required to establish the role of integrated smartwatch technology in important outcomes such as glycemic control, exercise participation, drug adherence, and diet monitoring in people with all forms of diabetes mellitus.

## Introduction

### Background

Mobile health (mHealth) solutions, which include mobile apps, have rapidly gained popularity as part of the overall management of chronic diseases and have further created opportunities and potential to enhance the ability for self-management in patients with diabetes mellitus (DM) and reduce long-term and irreversible health complications [1-3]. More recently, the introduction of smartwatch technology has opened up the opportunity for the increased use of these wearable devices for health and wellness [4], as well as for the management of chronic medical conditions, such as monitoring chronic obstructive pulmonary disease [5], and health outcomes, such as physical activity monitoring in the older adult population [6]. Smartwatch technology has also seen increasing adoption for use in common chronic conditions such as atrial fibrillation [7] and other cardiac arrhythmias. Most of the early smartwatch intervention research has focused on the accelerometer smartwatch data as a surrogate measure of exercise; however, as innovation in smartwatch technology evolves, more sources of data input become available [8].

Companies manufacturing smartwatches have focused on developing applications that address a broader range of general health and lifestyle interventions such as weight, diets, and exercise. Examples of popular applications include Sugar Sense, Grab Manager, and Diabetes Tracker; however, none have been evaluated for their effects on clinical endpoints. Traditionally, technological companies developing glucose monitoring devices have subsequently launched proprietary applications such as Freestyle Libre Link App and Dexcom Clarity for either personal computers or mobile devices. Henriksen et al [9] showed that research into the area of wrist-worn fitness wearable devices has been accelerating, and technology giants include companies such as Fitbit, Garmin, Misfit, Apple, and Polar, with possible applications in patient diagnostics and treatment. The lack of glucose monitoring technology in smartwatches has served as a real barrier to their broader usefulness in patients with diabetes. This has somewhat been addressed by mobile phones that can integrate data from stand-alone glucose monitoring devices, most recently continuous glucose monitoring (CGM), and other minimally invasive devices such as flash glucose monitoring (FGM). However, early attempts to develop technology such as the GlucoWatch G2 biographer, which was designed to detect trends and track patterns in glucose levels, had setbacks in commercial development [10].

The ability of smartwatch technology to display goals visually and offer motivational graphics upon completion of these goals should not be underestimated. These devices deliver behavioral influences that encourage patients to set specific daily, weekly, or monthly targets and achieve them. Wearable devices can also be wirelessly connected in real time to smartphones that record important outcome data, such as blood glucose levels, which are then automatically synchronized with a freely available and password-protected cloud network. This network is accessible live at any time and anywhere by the patient, family members, and clinicians, allowing potential multidisciplinary clinical decisions to be made in a much more integrated, informed, and prompt manner [11].

The aim of this systematic review is to explore the evidence for the effect and provision of smartwatch technology in patients with type 2 DM (T2DM). This review will also include studies exploring the use of this technology in type 1 DM (T1DM) and gestational DM (GDM).

### Research Questions

The following were our research questions:

1. What role can wearable smartwatch-based technology play in the provision of care and improvement of behavioral and clinical outcomes for patients with T1DM, T2DM, or GDM?
2. What is the acceptability and usability of smartwatch-based technology in the care of patients with DM?

## Methods

This review was registered with the PROSPERO (International Prospective Register of Systematic Reviews) database for systematic reviews (CRD42019136825).

### Search Strategy

The following databases were searched: PubMed, Ovid MEDLINE, Ovid Embase, Cochrane Library, Scopus, Web of Science (Social Sciences Citation Index and Science Citation Index Expanded), IEEEXplore, and CINAHL in August 2023. The search strategy was developed using keywords from systematic reviews that focus on smartwatch technology, mHealth, or mobile app–based interventions in patients with either T1DM, T2DM, or GDM. The OVID search strategy has been provided in Multimedia Appendix 1.

No restrictions were placed on the dates of articles or type of articles during the initial search. Specific search terms were adopted to reflect the requirements of each database. Thesaurus or MeSH (Medical Subject Headings) terms and truncation appropriate to each database were used. Furthermore, ProQuest Dissertations and Theses Global (plus full-text 1997-present) were searched for relevant dissertations. Theses and conference proceedings were searched via Scopus to capture any additional pertinent research in this emerging field. Reference lists of included studies, as well as the reference lists of related systematic reviews and meta-analyses, were searched to identify any relevant studies. If potential eligible articles were not in English, they were to be translated; however, this was not required.

### Types of Studies to Be Included

To capture the full extent of the research literature, any relevant quantitative research addressing the use of smartwatch technology in the treatment of DM was considered. Eligible study designs included randomized controlled trials (including randomized crossover studies and cluster randomized trials), quasi-experimental studies (including interrupted time series studies), controlled before and after studies and observational studies (cohort, case-control, and observational studies (cohort, case-control, and cross-sectional studies). Partially published work (eg, conference abstracts) was eligible only if the full-text reports could be identified.

### Participants/population

Participants of any age with a current or previous diagnosis of T1DM, T2DM, and GDM were included.

### Intervention(s) and Exposure(s)

Studies using smartwatch technology as an intervention for patients with T1DM, T2DM, or GDM were included. Both wrist-worn and clip-on smartwatches were included; however, they must be “smart devices” (Bluetooth or Wi-Fi enabled to allow synchronizing with a mobile phone app or website) and must be commercially available.

### Comparator(s)/control

Potential studies were not specifically excluded if they did not include a control group. Should studies include a comparator group, they may include alternative mHealth interventions, non–smartwatch app-based interventions, or standard-of-care or placebo interventions.

### Exclusion Criteria

The rationale for including nonrandomized studies and extension to studies including T1DM and GDM, beyond T2DM, was pragmatic, as a limited number of randomized clinical trials (RCTs) evaluating the use of smartwatch technology in the management of DM were identified. Studies evaluating patients only with prediabetes were excluded.

### Outcomes of Interest

#### Primary Outcomes

The primary outcomes were as follows:

1. Glycemic control: including measures of hemoglobin A_1c_ (HbA_1c_), CGM parameters using subcutaneous CGM or interstitial FGM, or measures of diabetic complications (eg, fetal or maternal outcomes in GDM).
2. Lifestyle and medication: at least one measure of either objective or subjective record of physical activity, dietary records, or medication adherence.

#### Secondary Outcomes

The secondary outcomes were acceptance, usability, and acceptability of the smartwatch as an intervention in patients with T1DM, T2DM, or GDM. This included study retention rates, how often patients wore the smartwatch, and any comments/qualitative data on the patient’s perception of the watch.

### Data Extraction

The PRISMA (Preferred Reporting Items for Systematic Reviews and Meta-Analyses; Checklist 1) guidelines were used in the identification of eligible studies. Of the studies retrieved from the initial search, titles and abstracts were screened by the authors (AF and DS) using Covidence software. Full texts of potential eligible studies were then screened by two independent authors (AF and DS) to identify eligible studies, with discrepancies resolved by a third reviewer (SDA) if required. The review assessed and discussed the effect of missing data and the degree of its effect on the overall synthesized results.

Data were extracted using a standardized form including the following parameters: publication details (authors, year, and country of study), participant characteristics (number of participants, baseline characteristics, inclusion and exclusion criteria, and type of diabetes), methods (study design, baseline measure, time points [when data were collected: at baseline and the end of the study], and study setting [location, year, and environment]), intervention duration and description including manufacturer/brand of smartwatch used, outcome measures used to identify the effects of the smartwatch intervention, smartwatch-related process evaluation outcomes (usability, acceptability, adherence, and interaction), measures of glycemic clinical outcomes and diabetic complications; and limitations of the study.

### Risk of Bias Assessment

The Downs and Black (1998) methodological quality assessment checklist was used as different types of study were anticipated [12]. Two independent authors (DS and AF) assessed the risk of bias, with discrepancies in checklist items resolved by the team; if resolution was not achieved, an arbitrator (AC) was nominated. This was never required.

After analysis, study outcomes were reported using summarized descriptive analysis, focusing on the types of study participants, types of interventions, types of smartwatches, length of follow-up, clinical and behavioral outcomes, and smartwatch-related results.

The overall quality of evidence for an outcome was assessed using a Grading of Recommendations, Assessment, Development, and Evaluations (GRADE) approach, which uses the summary of the risk of bias of the outcome across studies to assess the robustness of the evidence [13]. The GRADE approach uses assessments across 5 domains—study limitations, consistency of results, directness of the evidence, precision of the results, and publication/reporting bias—to categorize the levels of quality as high, moderate, low, and very low [13].

### Ethical Considerations

This study did not include human subjects research (no human subjects experimentation or intervention was conducted) and so did not require institutional review board approval.

## Results

### Overview

**Figure 1.**
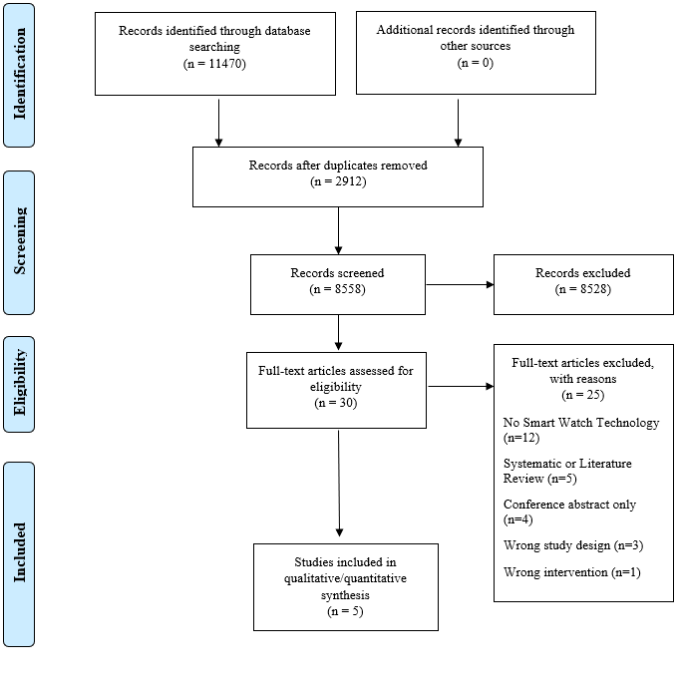
Depicts a Preferred Reporting Items for Systematic Reviews and Meta-Analyses flow diagram that was generated in Covidence software during the screening phases of this systematic review.

A total of 11,470 articles were retrieved from the database searches, of which 2912 were immediately removed as duplicates. Overall, 8558 articles were screened by the two independent reviewers; ultimately 30 studies were considered as potentially eligible, and full-text articles were retrieved. In general, 12 studies were excluded, as they did not incorporate smartwatch technology [14-25]. In total, 5 studies were systematic or literature reviews [26-30]. The reference lists of these reviews were screened for potentially eligible papers, but none were identified. There were 4 studies that were excluded, as they were conference abstracts only and a full-text paper was not available for adequate data extraction [31-34]. Among these studies, 3 studies were the wrong study designs, meaning they did not investigate the use of smartwatches or digital technology [35-37]. Finally, 1 study was excluded, as the intervention was not eligible for inclusion [38]. Multimedia Appendix 2 provides a list of excluded studies and the reasons for their exclusion. A total of 5 studies met the inclusion and exclusion criteria listed and were included for analysis in this review [11,39-42]. Figure 1 depicts the full PRISMA flow diagram and Table 1 provides the table of characteristics of included studies.

To date, 5 studies [11,39-42] have evaluated the integration of smartwatch technology in patients with a diagnosis of DM. Each included study’s results have been separately provided below due to the heterogeneous nature of methods and reporting.

**Table 1.** Characteristics of included studies.

Study	Population/sample/context	Aim/objectives	Technologies	Study design	Outcome measures	Results	Limitations
Årsand et al [11]	4 Participants with type 1 diabetes mellitus.Motol University Hospital, Prague.	Feasibility study to test the usability and acceptability of smartwatch technology in patients with type 1 diabetes mellitus.	Pebbles smartwatch on android+ Diabetes Diary App	A novel 6-participant cohort study designed to give the research group feedback on smartwatch technology.	Outcome measures focused on participant feedback on the technology as well as usability of the smartwatch and Diabetes Diary App	Authors concluded that overall users reported positive usability.Participants provided good feedback such as the user interface and being able to input measurements quickly and accessing them. Participants did suggest areas of improvement such as battery usage not able to delete inputs if added accidentally.	Limited sample size with no control group as study was novel and exploratory only. Short observation period.
Kim et al [39]	29 adults with type 2 diabetes mellitus.Seoul National University Hospital, South Korea.	Test the feasibility of HbA_1c_^a^ reduction using a patient-centered, smartphone-based, diabetes care system.	(1) Android-based application with four modules:glucose; diet; physical activity; and social network system(2) Bluetooth glucometer(3) Bluetooth activity tracker	12-week feasibility pilot study. One-arm group.	HbA_1c_, fasting plasma glucose, body weight,blood pressure, various cholesterol measures. Summary of diabetes self-care activities (SDSCA) was used to evaluate the overall self-management activities for diabetes	After 12 weeks, participants had significantly decreased HbA_1c_ and FPG^b^.Reduction in HbA_1c_ was correlated with the number of daily glucometer inputs. Inputs were generally higher in older patients.Body weight and cholesterol measures were not statistically significant after 12 weeks.	Pilot study. No control group.Short observation time. Small sample size.
Shaw et al [40]	60 adults with type 2 diabetes mellitus.South Eastern United States.	To determine feasibility and acceptability of using multiple mHealth technologies in patients with type 2 diabetes mellitus.	(1) Glucometer – “iHealth”(2) Fitbit(3) Self-report mobile SMS text messaging(4) Cellular enabled Scale by Body Trace	6-month cohort prospective study.	(1) Blood glucose(2) Physical activity – daily steps, distance travelled, and activity intensity(3) Medication adherence(4) Weight	mHealth interventions not used as to improve outcomes listed.Most used technology was the Fitbit.Participants who were younger, had higher HbA_1c_, and identified as Black were less likely to be engaged with their mHealth devices.	Only observational study. Did not use control group for interventional impact.Small sample size.
Zahedani et al [41]	665 participants: healthy (448); prediabetic (25); type 2 diabetics (192)	Investigate combined use of continuous glucose monitoring and mobile app (Sugar AI) on glucose tracing, heart rate, and physical activity.	(1) Abbott Freestyle Libre(2) MiBand 3 or Garmin watch(3) Sugar Artificial intelligence app	10-day observational study.	(1) Blood glucose, measured as TIR^c^: 54‐140 mg/dL for healthy andprediabetes, and 54‐180 mg/dL for type 2 diabetes mellitus	Authors concluded that a subgroup of those showing poor TIR (combined type 2 diabetes mellitus and prediabetic participants) demonstrated an average of 22.7% improvement in TIR.62.9% of diabetic participants who showed improved TIR had greater improvement in their daily variation.	Only observation study. Not randomized clinical trial. Short follow-up.Limited results provided on use of Garmin watch or MiBand 3 to improve outcome measures such as blood glucose levels or heart rate, etc.
Chang et al [42]	35 adults with type 2 diabetes mellitus.New South Wales, Australia.	To test the feasibility of prescribing an individualized daily exercise time.	(1) Abbott Freestyle Libre 2(2) Accelerometer smartwatch (ActiGraph Bluetooth Smart wGT3X-BT	Two-week observational period.	(1) Exercise adherence–feasibility. Proportion of participants completing more than 15 minutes of physical activity (moderate to high intensity)(2) Continuous glucose monitoring(3) Dietary intake	Authors found that participants increased their daily physical activity by an average of 10 minutes per day.However, authors did report adherence issues with a participants not conducting physical activity in the prescribed time.	Proof-of-concept randomized clinical trial. Small sample size. Short observational period.

a HbA_1c_: hemoglobin A_1c_.

b FPG: fasting plasma glucose.

c TIR: time in range.

### Årsand et al 2015

The “Pebble” smartwatch was tested on 4 participants with T1DM ranging from ages 20 to 46 years. Usability scores for this study averaged 4.4/5, with 5 being the best score. The highest scoring question was that all values were clearly shown on the smartwatch (4.8/5) and the lowest was the ability to make a new registration with the “Pebble” app (4.3/5). Qualitative feedback for appreciated features included the ability to see the last values and user interface while nonappreciated feedback included battery usage, not being able to delete entries from the watch, and the possibility of entering wrong values. A total of 2 of the 4 participants preferred to use their phone to input data as they found it more convenient [11].

### Kim et al 2016

An “LG LifeGram” activity tracker combined with a mobile-centered mobile app was tested on 29 participants with T2DM over 12 weeks. The intervention focused on delivering information on glucose control, diet, exercise, and finally a social support network for motivation. After 12 weeks, participants experienced a statistically significant reduction in HbA_1c_ (*P*=<.0001 with a decrease of 0.6%) and fasting plasma glucose (*P*=.0088 with a decrease of 20.8 mg/dL) levels. No changes were observed in body weight and cholesterol levels. Authors reported high input scores that correlated with the amount of change in HbA_1c_ levels (*P*=.0013) [39].

### Shaw et al 2020

A total of 60 adults with T2DM were observed for 6 months while using the “Fitbit Alta” (Google Fitbit), which is a reliable and valid accelerometer used for tracking physical activity. This smartwatch was paired with the participant’s smartphone app. A total of 87.45% of daily physical activity submissions for the “Fitbit Alta” were completed by participants, which was the highest achieving outcome for input completions. Other outcomes such as glucose monitoring (60.99%), drug adherence (71.19%), and weight (53.83%) did not score as high as physical activity and, interestingly, were not tracked using smartwatch technology. Technical support was also monitored during this study in which authors reported that 35/60 (58%) participants required assistance due to devices not syncing or logging-in problems [40].

### Zahedani et al 2021

The “Sugar AI” app was used in conjunction with a “MiBand 3” or “Garmin” in 192 participants with T2DM. It is noted that this study also involved healthy individuals and participants with self-diagnosed “prediabetes.” The primary outcome in this study was time in range that was defined as individuals with T2DM to be between 3 and 10 mmol/L. The authors reported that 58.3% (112/192) of participants with T2DM and a poor time in range showed an average improvement of 22.7%. Authors did not report adherence, feasibility, or adherence to the intervention [41].

### Chang et Al 2023

In this double-blinded proof-of-concept study, 35 participants with physician-diagnosed T2DM were issued a Freestyle Libre 2 (Abbott) and an “ActiGraph Bluetooth Smart wGT3X-BT.” These technologies were used and worn for a 14-day baseline period followed by a 14-day interventional period with the purpose of testing the feasibility of prescribing an exercise time to target peak hyperglycemia. The participants were further randomized into the “ExPeak” group, which aimed at conducting physical activity for 30 minutes before peak hyperglycemia, or the “NonPeak” group, which conducted physical activity for 90 minutes after peak hyperglycemia. Adherence to prescribed exercise times showed that only 29% (10/35) of participants adhered to their prescribed times. Overall, 66% (23/35) of participants completed their exercises but not during the times recommended to target peak hyperglycemia. the authors concluded that participation in moderate physical activity increased by 26% during the intervention period versus the baseline period but was not statistically significant between groups (*P*=.26). There was no statistically significant difference in the intensity of physical activity between groups (*P*=.77). Authors did not provide specific data on the usability of the smartwatch technology used in this study.

Data extraction revealed the heterogeneous nature of the studies included in this review. This extended to the risk of bias assessment, with results presented in Table 2. Only one study attempted to blind participants to their intervention and randomized this process with robust methods [42].

**Table 2.** Risk of bias of included studies.

Items	Årsand et al [11]	Kim et al [39]	Shaw et al [40]	Zahedani et al [41]	Chang et al [42]
1. Is the hypothesis/aim/objective of the study clearly described?	1^a^	1	1	1	1
*2.* Are the main outcomes to be measured clearly described in the *Introduction* or *Methods* section?	0^b^	1	1	1	1
3. Are the characteristics of the patients included in the study clearly described?	0	1	1	1	1
4. Are the interventions of interest clearly described?	1	1	1	1	1
5. Are the distributions of principal confounders in each group of subjects to be compared clearly described?	0	1	1	1	0 (UTD)^c^
6. Are the main findings of the study clearly described?	0	1	1	1	1
7. Does the study provide estimates of the random variability in the data for the main outcomes?	0	1	1	1	1
8. Have all important adverse events that may be a consequence of the intervention been reported?	0	1	0	0	1
9. Have the characteristics of patients lost to follow-up been described?	1	1	1	1	1
10. Have actual probability values been reported (eg, 0.035 rather than <0.05) for the main outcomes except where the probability value is less than 0.001?	0	1	1	1	1
11. Were the subjects asked to participate in the study representative of the entire population from which they were recruited?	0	0 (UTD)	0 (UTD)	1	0 (UTD)
12. Were those subjects who were prepared to participate representative of the entire population from which they were recruited?	0	0 (UTD)	0 (UTD)	1	0 (UTD)
13. Were the staff, places, and facilities where the patients were treated, representative of the treatment the majority of patients receive?	0 (UTD)	0	0	1	1
14. Was an attempt made to blind study subjects to the intervention they have received?	0	0	0	0 (UTD)	1
15. Was an attempt made to blind those measuring the main outcomes of the intervention?	0	0 (UTD)	0 (UTD)	0 (UTD)	1
16. If any of the results of the study were based on “data dredging,” was this made clear?	0 (UTD)	0	0 (UTD)	0 (UTD)	0 (UTD)
17. In trials and cohort studies, do the analyses adjust for different lengths of follow-up of patients, or in case-control studies, is the time period between the intervention and outcome the same for cases and controls?	0 (UTD)	1	1	1	1
18. Were the statistical tests used to assess the main outcomes appropriate?	1	1	1	1	1
19. Was compliance with the intervention(s) reliable?	1	1	1	0	0
20. Were the main outcome measures used accurate (valid and reliable)?	0 (UTD)	1	1	1	1
21. Were the patients in different intervention groups (trials and cohort studies) or were the cases and controls (case-control studies) recruited from the same population?	0 (UTD)	1	1	1	0 (UTD)
22. Were study subjects in different intervention groups (trials and cohort studies) or were the cases and controls (case-control studies) recruited over the same period of time?	0 (UTD)	1	1	0 (UTD)	1
23. Were study subjects randomized to intervention groups?	0	0	0	0	1
24. Was the randomized intervention assignment concealed from both patients and health care staff until recruitment was complete and irrevocable?	*0*	*0*	0	0	1
25. Was there adequate adjustment for confounding in the analyses from which the main findings were drawn?	0 (UTD)	0 (UTD)	0 (UTD)	0 (UTD)	0 (UTD)
26. Were losses of patients to follow-up taken into account?	1	1	1	1	1
27. Did the study have sufficient power to detect a clinically important effect where the probability value for a difference being due to chance is less than 5%?	0	0 (UTD)	0	0 (UTD)	0 (UTD)
Total score	6	17	16	17	19

a 1: yes.

b 0: no.

c 0 (UTD): unable to determine.

## Discussion

### Principal Findings

This systematic review found that among the current literature, only 5 studies have explored the use of smartwatch technology in people with DM (T1DM, T2DM, and GDM). A total of 4 of the 5 included studies recruited participants with T2DM, while no studies included participants with GDM. This review also provides evidence of the paucity of robust RCTs that evaluate the effect of smartwatch technology in the management of patients diagnosed with DM. Recent research developments have evaluated transcutaneous noninvasive sampling technology such as noninvasive optical glucose monitoring, based on optical glucose monitoring, and noninvasive fluid sampling, based on fluid sample glucose estimation, but these are in their infancy [43]. These technologies aim to reduce the need for patients with diabetes to use the more traditional finger-prick method of measuring their blood glucose levels, which can be painful, more time-consuming, and reduce compliance [44]. It may be an additional impetus for technology development if smartwatches are able to harness these technologies to monitor patients’ glucose effectively and noninvasively. T1DM is the area that is most suitable for the development of smartwatch technology for the management of diabetes. Patients with T1DM tend to be younger and thus often have more agile technological literacy and are often early adopters of technological developments. T1DM is also a condition prone to hypoglycemia and thus an important area for technologies that may reduce the risk of developing serious complications that may arise due to hypoglycemia unawareness, which may be where smartwatch technology can most appropriately intercept in patients that exhibit a lack of warning symptoms [45]. The same could also be said for patients with hyperglycemia and life-threatening diabetic ketoacidosis. The carers of patients with T1DM also value the ability to use technology for distant monitoring of children and adolescents with T1DM. Emerging evidence on smartwatch technology may be available soon, with Sehgal et al [46] recently publishing a protocol that will assess the safety and efficacy of the addition of smartwatch technology to usual CGM in adults with T1DM. The authors have stated the primary endpoint will be glucose time in range expressed as a percentage time interstitial glucose between 3.9 and 10 mmol/L. Other outcomes will include quality of life, distress, and sleep quality [46]. Finally, Corbett et al [47] published data on the use of smartwatch gesture–based meal reminders using a proprietary app that picks up “eating motions” by using the smartwatch and uses this to remind patients with T1DM to inject insulin if appropriate.

DM is a heterogenous condition and there is provision for future research in the use of smartwatch technology in all types of diabetes. There is an opportunity for this technology to combine with others like the FreeStyle Libre 2 to prompt regular blood glucose level monitoring, improve time in range and variability, and reduce the risk of serious complications such as hypoglycemia. Moreover, as more evidence emerges on other subtypes of DM such as diabetes of the exocrine pancreas (type 3c), the provision for evidence-based technologies to monitor and improve outcomes increases [48]. Detailed usability and acceptability trials followed by robust long-term clinical trials should be conducted to test these emerging smart technologies prior to their introduction into the management of patients with diabetes.

### Limitations of the Study

This review does not include quantitative analyses or meta-analyses due to a lack of homogenous RCTs. Most papers included were feasibility, usability, and/or acceptability studies and did not include a comparator group. The quality of the papers included was sound. Improvements in blinding of participants and team members to intervention groups need to be considered in future research as well as the sampling of patients selected for studies to promote generalizability of results. This is an area that is rapidly developing and even though an attempt has been made to encompass all contemporaneous literature available, there may be new evidence that will inform the topic. Technology is rapidly developing within the commercial and proprietary arena, making it unavailable for systematic review.

### Conclusion

This systematic review highlights the current paucity of evidence supporting the use of smartwatch technology in the management of patients with all types of DM. As smartwatch usage increases with greater affordability and comfort with the technology grows, so does the potential for their significant role in the management of DM. This would especially be the case if wearable technology seamlessly interfaces with glucose monitoring devices or can serve to monitor glucose directly through innovative developments in noninvasive glucose monitoring. Smartwatches would allow patients the ability to check their glucose level frequently and discretely if they are linked to a CGM device. Nutrition and physical activity are cornerstones of diabetes management. Mobile technologies, such as smartphones and wearables, have the potential to educate, motivate, and prompt individuals to optimize lifestyle interventions and thereby favorably affect health.

## Supplementary material

10.2196/54826 Multimedia Appendix 1 Search strategy.

10.2196/54826 Multimedia Appendix 2 Excluded studies.

10.2196/54826 Checklist 1 PRISMA (Preferred Reporting Items for Systematic Reviews and Meta-Analyses) checklist.

## Abbreviations

CENTRAL: Cochrane Central Register of Controlled Trials
CGM: continuous glucose monitoring
DM: diabetes mellitus
GDM: gestational diabetes mellitus
GRADE: Grading of Recommendations, Assessment, Development, and Evaluations
HbA_1c_: hemoglobin A_1c_
MeSH: Medical Subject Headings
mHealth: mobile health
PRISMA: Preferred Reporting Items for Systematic Reviews and Meta-Analyses
PROSPERO: International Prospective Register of Systematic Reviews
RCT: randomized clinical trial
T1DM: type 1 diabetes mellitus
T2DM: type 2 diabetes mellitus
