# Supplementary material for: The Role of Smartwatch Technology in the Provision of Care for Type 1 or 2 Diabetes Mellitus or Gestational Diabetes: Systematic Review
Source: JMIR Mhealth Uhealth. 2024 Dec 3;12:e54826. doi: 10.2196/54826 (PMC11629918; doi:10.2196/54826)
Supplement: Multimedia Appendix 2 [file mhealth-v12-e54826-s002.docx]

| Supplementary Table 2. Table of Excluded Studies | | |
| --- | --- | --- |
| Reference | Author | Reason for Exclusion |
| [14] | Garg et al 2017 | Excluded due to participants not being issued SmartWatch technology. |
| [15] | Wang et al 2020 | Excluded due to outcome of study not relevant to review question and no SmartWatch technology. |
| [16] | Hirst et al 2014 | Excluded due to participants not being issued SmartWatch technology. |
| [17] | Keith-Hynes et al 2014 | Excluded due to participants not being issued SmartWatch technology. |
| [18] | Asante et al 2020 | Excluded due to participants not being issued SmartWatch technology. |
| [19] | Zhai et al 2020 | Excluded due to participants not being issued SmartWatch technology. |
| [20] | Poulter et al 2022 | Excluded due to participants not being issued SmartWatch technology. |
| [21] | Pamungkas et al 2022 | Excluded due to participants not being issued SmartWatch technology. |
| [22] | Yang et al 2020 | Excluded due to participants not being issued SmartWatch technology. |
| [23] | Yu et al 2019 | Excluded due to participants not being issued SmartWatch technology. |
| [24] | Gunawardena et al 2019 | Excluded due to participants not being issued SmartWatch technology. |
| [25] | Garnweidner-Holme et al 2020 | Excluded due to participants not being issued SmartWatch technology. |
| [26] | Martos-Cabrera et al 2020 | Excluded due to being a systematic review. Reference list screened for additional eligible papers. |
| [27] | Kerr et al 2019 | Excluded due to being a literature review. |
| [28] | Hartz et al 2016 | Excluded due to being a systematic review. Reference list screened for additional eligible papers. |
| [29] | Kebede et al 2018 | Excluded due to being a systematic review. |
| [30] | Constantin et al 2018 | Excluded due to being a systematic review. |
| [31] | Al-Haijri etl al 2020 | Excluded due to being a conference abstract and review. |
| [32] | Yang et al 2016 | Excluded due to being a conference abstract only. Full text paper (Yang et al 2020) excluded. |
| [33] | Poster Abstract | Excluded due to incorrect citation. Poster abstract only. |
| [34] | Poster Abstract | Excluded due to incorrect citation. Poster abstract only. |
| [35] | Fleming et al 2020 | Excluded due to being a consensus report by EASD. |
| [36] | Kebede et al 2019 | Excluded due to being a survey (study design) |
| [37] | Cappon et al 2022 | Excluded due to wrong study design. |
| [38] | DirecNet 2003 | Excluded due to wrong intervention. |
